# Relationship Between Thyroid Hormonal Function and Ultrasound TI-RADS Stratification in a Saudi Cohort

**DOI:** 10.3390/metabo16030165

**Published:** 2026-02-28

**Authors:** Ali H. Alghamdi, Ashwag A. Albalawi, Shahad S. Aljuhani, Ahmed Alghamdi, Mansuor A. Alanazi, Arwa Baeshen, Adnan Alahmadi, Njoud Aldusary

**Affiliations:** 1 Department of Radiological Sciences, Faculty of Applied Medical Sciences, University of Tabuk, Tabuk 71491, Saudi Arabia; 2 Department of Internal Medicine, King Fahad Specialist Hospital, Tabuk Health Cluster, Tabuk 47717, Saudi Arabia; ashwaka@moh.gov.sa (A.A.A.); shsualjuhani@moh.gov.sa (S.S.A.); 3 Department of Endocrinology, King Fahad Specialist Hospital, Tabuk Health Cluster, Tabuk 47717, Saudi Arabia; aalghamdi572@moh.gov.sa; 4 Department of Family and Community Medicine, Faculty of Medicine, University of Tabuk, Tabuk 71491, Saudi Arabia; menazi@ut.edu.sa; 5 Radiological Sciences Department, College of Applied Medical Sciences, King Saud University, Riyadh 11433, Saudi Arabia; abaeshen@ksu.edu.sa; 6 Radiologic Sciences, Faculty of Applied Medical Sciences, King Abdulaziz University, Jeddah 21589, Saudi Arabia; aaalahmadi@kau.edu.sa (A.A.); naldossary@kau.edu.sa (N.A.)

**Keywords:** thyroid nodules, thyroid ultrasound, TI-RADS, thyroid-stimulating hormone (TSH), free thyroxine (FT4), thyroid function tests, ultrasound risk stratification, cross-sectional study

## Abstract

Introduction: Thyroid disorders are among the most prevalent endocrine diseases worldwide, with rising incidence linked to aging, lifestyle, and environmental factors. Early identification of both functional and structural abnormalities is essential to prevent complications. This study aimed to investigate the coherence between thyroid function as measured by thyroid-stimulating hormone (TSH) and free thyroxine (FT4) tests and ultrasound-based thyroid classification according to the American College of Radiology Thyroid Imaging Reporting and Data System (ACR TI-RADS). Methods: This retrospective cross-sectional study included patients in Tabuk, Saudi Arabia, who underwent thyroid ultrasound alongside TSH and FT4 tests within two weeks. Thyroid nodules were classified using TI-RADS. Demographic, clinical, and laboratory data were extracted from electronic records, and statistical analyses examined associations between hormone levels, ultrasound findings, and clinical variables (*p* < 0.05). Results: A total of 102 patient records were included in the study. Most participants were female and overweight, with a mean body mass index (BMI) of 30.2 ± 4.6 kg/m^2^. The majority were euthyroid (58.3%) or subclinical hypothyroid (27.8%); most nodules were benign (TI-RADS 2–3). BMI showed a moderate positive correlation with TSH (ρ = 0.20, *p* = 0.041) and a negative correlation with FT4 (ρ = –0.20, *p* = 0.040). No significant relationship was observed between TI-RADS classification and thyroid hormone levels (*p* > 0.05). Conclusions: Structural thyroid changes identified by ultrasound appeared largely independent of hormonal status. Meanwhile, BMI demonstrated a modest physiological association with thyroid function reflected in TSH and/or FT4 levels. These findings emphasize the need for integrated biochemical and imaging evaluation to enhance diagnostic precision in the assessment of thyroid disease.

## 1. Introduction

The global prevalence of thyroid dysfunction—including both hypo- and hyperthyroidism—has risen steadily in recent decades, with estimates ranging from 5% to 30% depending on region and diagnostic criteria [1]. This increase has been variously attributed to aging populations, environmental factors, and advances in diagnostic tools, such as ultrasound (US) imaging technology [2,3,4]. Structural abnormalities, such as thyroid nodules, are also highly frequent, detected in up to 60% of adults when sensitive ultrasonography is applied [5,6]. Although most nodules are benign, approximately 5–10% harbor malignancy, emphasizing the clinical need for precise risk stratification [7,8].

The thyroid gland secretes thyroxine (T_4_) and triiodothyronine (T_3_), under the control of thyroid-stimulating hormone (TSH), which play central roles in metabolic regulation, thermogenesis, cardiovascular performance, and neurocognitive function. Dysregulation of this axis—whether biochemical or structural—can have multisystemic consequences. Hypothyroidism can lead to dyslipidemia, fatigue, and cognitive decline, whereas hyperthyroidism may cause arrhythmia, bone loss, and muscle weakness [1]. Because such manifestations are often subtle, the early detection of thyroid dysfunction and nodular changes is crucial for preventing irreversible complications and improving quality of life [5,9].

US is generally preferred for thyroid imaging due to its minimal or absent risk, high sensitivity, non-invasive nature, and ability to detect small lesions invisible to palpation. The American College of Radiology Thyroid Imaging Reporting and Data System (ACR TI-RADS) assigns malignancy-risk categories based on various criteria—including echogenicity, margins, and calcifications—standardizing sonographic assessment [10]. This approach enhances diagnostic accuracy and minimizes unnecessary fine-needle aspirations (FNA) [11]. US-based risk stratification systems are primarily driven by structural imaging features; while thyroid hormone levels also reflect functional status, the relationship between structural and biochemical markers remains incompletely understood. Future studies that combine biochemical and imaging data are therefore necessary.

In Saudi Arabia, thyroid disorders reflect global patterns but occur with higher prevalence, especially among women. Community-based and hospital studies indicate that between 25–40% of Saudi individuals exhibit some kind of thyroid disease, with subclinical hypothyroidism being the most prevalent among participants [12,13]. Recent records reveal that thyroid cancer ranks among the top three malignancies nationwide and is the second most common cancer in women [14]. Research in several regions shows that iodine-deficient areas persist in southern and western regions despite salt iodization initiatives, leading to endemic goiter and nodular illness [15]. Together, these findings highlight a regional need for precise, integrative diagnostic frameworks that combine laboratory and imaging markers.

Several studies have explored the measurement of serum TSH as a potential predictor of malignancy in thyroid nodules. However, most have relied on cytological or histopathological outcomes, rather than standardized US-based risk stratification systems such as TI-RADS. As a result, whether thyroid hormone status is consistently associated with imaging-defined risk categories, independent of cytological confirmation, remains uncertain. In addition, evidence is limited in Middle Eastern populations, where patterns of iodine intake and the prevalence of autoimmune thyroid disease may differ. This highlights an important knowledge gap regarding whether biochemical thyroid function meaningfully corresponds with US-based nodule risk in routine clinical practice.

Accordingly, the present study aims to investigate the relationship between thyroid hormone levels—specifically TSH and free T_4_ (FT4)—and anatomical US features of thyroid nodules as classified by TI-RADS among patients attending an endocrinology clinic in Tabuk, Saudi Arabia. By retrospectively analyzing records of real-world data, this study seeks to determine whether thyroid hormonal status is independently associated with standardized US risk stratification, addressing a gap between biochemical and imaging-based approaches in routine thyroid evaluation.

## 2. Methods

### 2.1. Study Design

This retrospective cross-sectional study was conducted at a single tertiary hospital in Tabuk, Saudi Arabia. We examined the relationship of TSH and free T_4_ with thyroid nodule features identified with US and classified according to TI-RADS. Data were extracted for patients evaluated between January 2025 and December 2025 from the endocrinology department’s electronic medical records.

### 2.2. Participants

The study population consisted of adult patients aged 18 years or older who had undergone both thyroid US and laboratory assessment of TSH and FT4. To ensure temporal proximity between functional and structural assessments, only patients with TSH and FT4 measurements performed within two weeks of the US examination were included. Only the first available record for each patient was included to avoid duplication. Only patients with complete clinical and imaging documentation were eligible, and the US must have been performed specifically for thyroid nodule evaluation. Patients with a history of thyroid surgery or radioiodine ablation, those currently receiving thyroid hormone replacement or anti-thyroid medications, and those with incomplete or inconsistent laboratory or imaging records were excluded. Pregnant patients and individuals with other known endocrine disorders that could influence thyroid function were also excluded from the analysis. The final dataset therefore represented a clinically stable outpatient population undergoing diagnostic thyroid evaluation.

### 2.3. Ethical Approval

The study protocol was reviewed and approved by the Institutional Review Board (IRB) of the University of Tabuk (UT-782-346-2025) and facilitated by the Research Ethics Committee of King Fahad Specialist Hospital, Tabuk. Given the retrospective nature of the research, the requirement for informed consent was waived. All data were anonymized prior to analysis, and the study was conducted in accordance with the ethical standards of the Declaration of Helsinki.

### 2.4. Data Collection

Data were extracted from the hospital’s electronic database and radiology information system. Serum TSH and FT4 levels were measured using the Abbott Alinity ci-series integrated immunoassay and clinical chemistry system (Abbott Laboratories, Abbott Park, IL, USA), in accordance with the manufacturer’s instructions. Thyroid US examinations were performed using a high-resolution US system (Aplio i800, Canon Medical Systems Corporation, Tochigi, Japan) equipped with a high-frequency linear transducer, in accordance with standard thyroid imaging protocols. The nodule count, size, and TI-RADS categorization were noted for the most suspicious nodule of each patient. All US examinations were interpreted by board-certified radiologists with at least five years of experience in thyroid imaging. TI-RADS classification was applied according to ACR guidelines as part of routine institutional reporting practice. Although no formal interobserver agreement analysis was performed, image interpretation followed standardized reporting criteria within the department. Additionally, demographic information was gathered from patient records, including age, sex, and body mass index (BMI).

Thyroid autoimmunity markers (anti-thyroid peroxidase [anti-TPO] and anti-thyroglobulin [anti-Tg] antibodies) were not consistently measured in routine evaluation and were therefore excluded from the analysis.

### 2.5. Data Analysis

IBM SPSS Statistics version 26.0 (IBM Corp., Armonk, NY, USA) was used for statistical analysis. Given the retrospective design and use of routinely collected clinical records, an a priori sample size calculation was not performed. The final sample size was determined by the number of eligible patients meeting the inclusion criteria during the study period. Participant characteristics were summarized using descriptive statistics, including means, standard deviations, and frequency distributions.

Categorical variables were summarized using frequencies and percentages. Associations between categorical variables such as TI-RADS category and fine-needle aspiration (FNA) cytology results were examined using the chi-squared (χ^2^) test. Where expected cell counts were small, results were interpreted with caution due to limited statistical power. A two-tailed *p*-value < 0.05 was considered statistically significant.

The Shapiro–Wilk test was applied to assess data normality. Depending on the data distribution, Spearman correlation coefficients were calculated to explore associations between thyroid hormone levels (TSH and FT4), TI-RADS scores, and the number of nodules. Group differences in thyroid function categories and TI-RADS classifications were explored using the Kruskal–Wallis test or ANOVA. To examine whether TI-RADS category was independently associated with thyroid hormone levels, multivariable regression models were constructed with TSH and FT4 as outcome variables. Because TSH exhibited a right-skewed distribution, it was log-transformed prior to analysis. The models were adjusted for age, sex, and BMI. Robust standard errors were applied to improve the reliability of the estimates.

## 3. Results

### 3.1. Participant Characteristics

**Figure 1:**
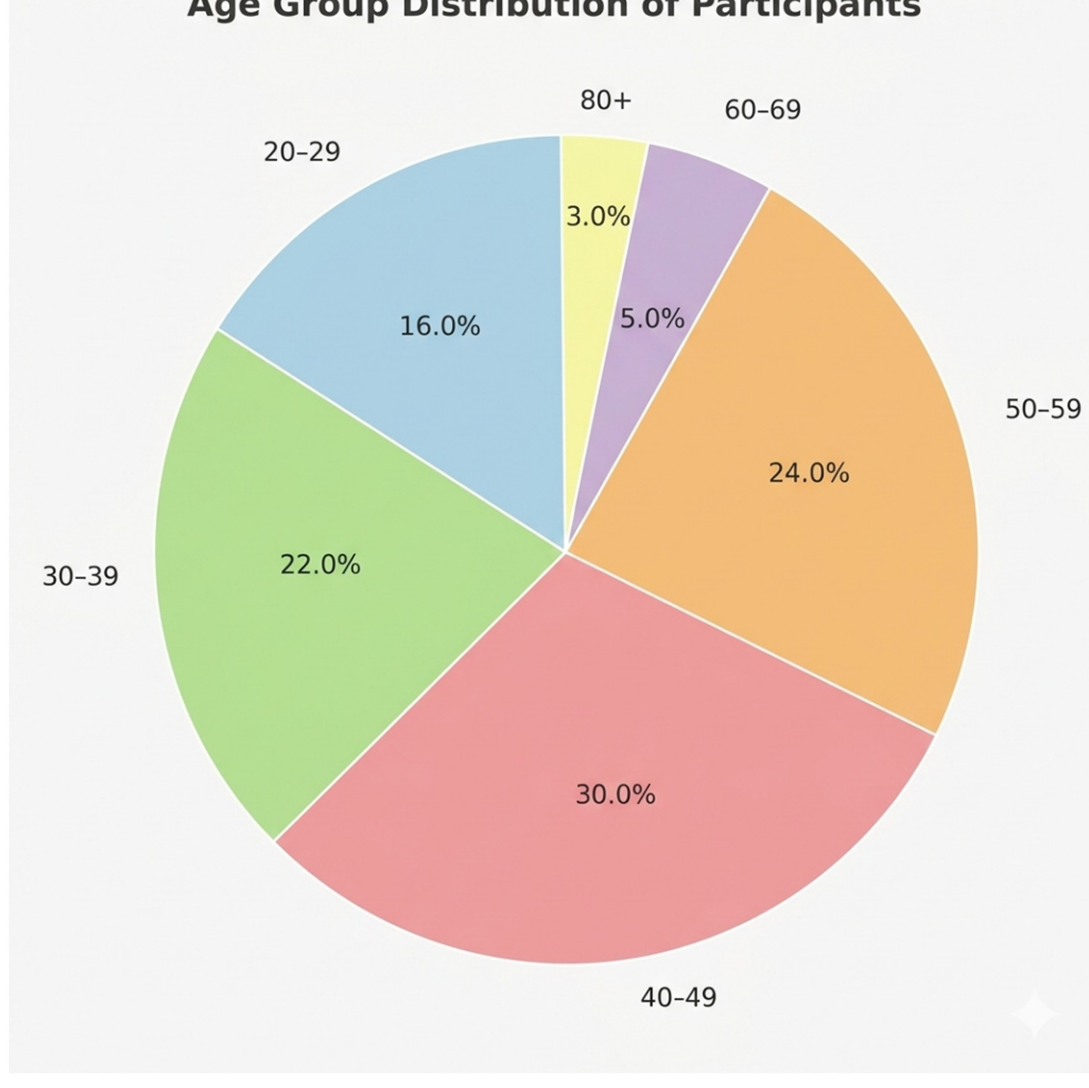
Age group distribution of participants. The majority of individuals were between 30 and 59 years old, accounting for more than half of the sample. A smaller proportion were in younger and older age categories.

A total of 102 patients aged 18 to 80 years were included in the analysis, of whom 76.5% (*n* = 78) were female, and 23.5% (*n* = 24) were male. Most participants were between 30 and 59 years, with a smaller proportion in the younger and older age categories (Figure 1). The average BMI fell in the overweight to obese range (Table 1), consistent with typical patterns reported in thyroid clinic populations.

### 3.2. Distribution of Thyroid Function and Ultrasound Findings

**Figure 2:**
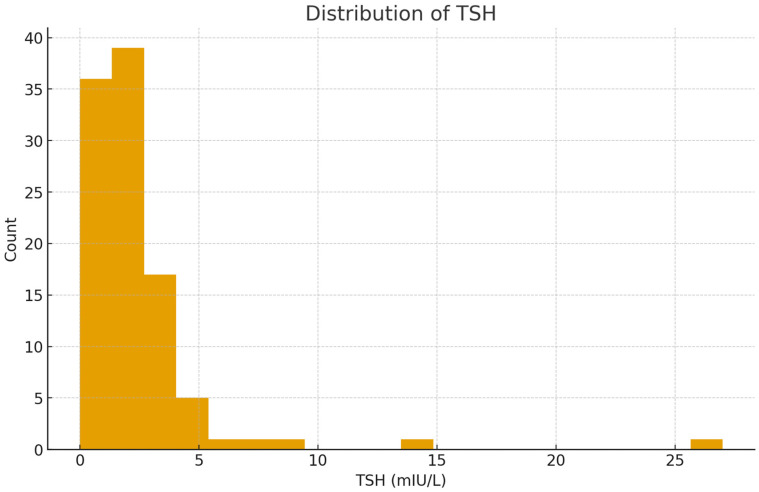
Distribution of thyroid-stimulating hormone (TSH) levels among participants. Most values clustered within the normal reference range (0.4–4.0 mIU/L), while a smaller number of individuals exhibited elevated TSH, indicating mild or overt hypothyroid tendencies. The right-skewed pattern reflects the infrequent occurrence of high TSH levels, consistent with typical population data for thyroid function testing.

**Figure 3:**
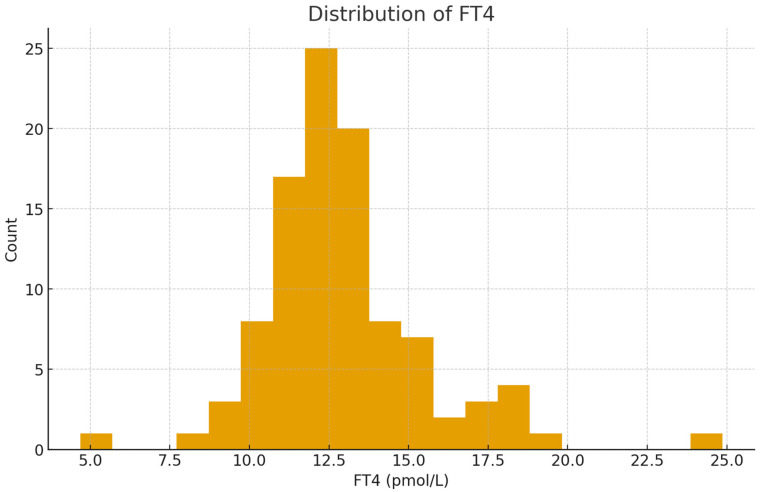
Distribution of free thyroxine (FT4) levels among participants. The majority of FT4 values were concentrated within the normal physiological range (approximately 10–23 pmol/L), with a near-normal distribution. Few participants showed markedly low or high FT4 concentrations, reflecting stable thyroid hormone production for most individuals in the study, consistent with euthyroid or subclinical states.

The mean TSH level was within the reference range, but varied widely among individuals; meanwhile, FT4 showed a narrower distribution (Figure 2 and Figure 3). The number of nodules detected in each patient varied between one and three. The majority of US findings were classified as TI-RADS 2 or 3, indicating that the nodules generally appeared benign.

### 3.3. Correlations Among Clinical and Hormonal Variables

Spearman correlation analysis revealed several statistically significant relationships. Age and BMI were moderately correlated (ρ = 0.41, *p* < 0.001), indicating that body mass tended to increase with age. BMI also showed a weak but significant positive correlation with TSH (ρ = 0.20, *p* = 0.041) and a negative correlation with FT4 (ρ = –0.20, *p* = 0.040). These results suggest a slight trend toward higher TSH and lower FT4 values as body mass increases, consistent with earlier findings that link obesity with altered thyroid activity.

**Figure 4:**
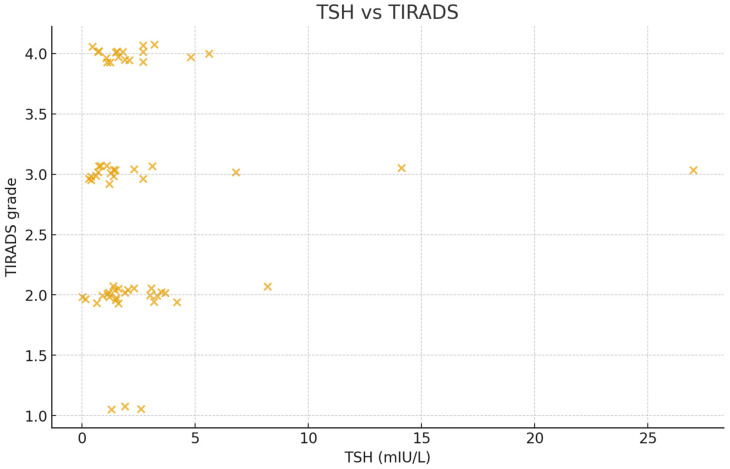
Relationship between thyroid-stimulating hormone (TSH) levels and Thyroid Imaging Reporting and Data System (TI-RADS) grades. Most participants clustered within normal TSH levels (0.4–4.0 mIU/L) and lower TI-RADS categories (2–3), indicating predominantly benign ultrasound features. A few cases with elevated TSH showed slightly higher TI-RADS scores, suggesting a possible but weak trend between thyroid functional changes and nodule risk category. Overall, no strong linear correlation was observed.

No significant correlation was observed between TI-RADS scores and either TSH or FT4 (each *p* > 0.05). Similarly, no meaningful relationship was found between the number of nodules and thyroid hormone levels. This indicates that the presence or quantity of nodules was not directly connected to thyroid function in this group of participants. However, descriptive patterns indicated the tendency for slightly higher TI-RADS categories among individuals with marginally elevated TSH; however, this association was not statistically significant (Figure 4).

### 3.4. Cytology (Fine-Needle Aspiration)

FNA data were available for only 22 patients; it was not requested for other patients in the sample. The majority of these samples were reported as benign lesions (e.g., colloid or adenomatoid nodules), while a few were suspicious or malignant. A chi-squared test examining the relationship between FNA category and TI-RADS score approached statistical significance but did not reach the predefined threshold (χ^2^(4) = 8.76, *p* = 0.068). This pattern suggests that higher TI-RADS grades were generally linked to more suspicious cytology findings (Table 2, Figure 4).

No significant differences were observed in Kruskal–Wallis tests comparing TSH and FT4 levels across different FNA categories (*p* = 0.27 and 0.60, respectively). However, most benign lesions were associated with hormone levels within the normal range.

### 3.5. Associations Between Thyroid Status and Other Variables

#### 3.5.1. Distribution of Thyroid Function Categories

Participants were classified into five categories of thyroid function based on standard international guidelines and their corresponding hormone levels (Table 3 and Table 4).

The majority of participants sampled were euthyroid (58.3%) or had subclinical hypothyroidism (27.8%); subclinical hyperthyroidism (7.4%), overt hypothyroidism (4.6%), and overt hyperthyroidism (1.9%) were less common.

#### 3.5.2. Associations of Thyroid Status with Demographic and Anthropometric Variables

**Figure 5:**
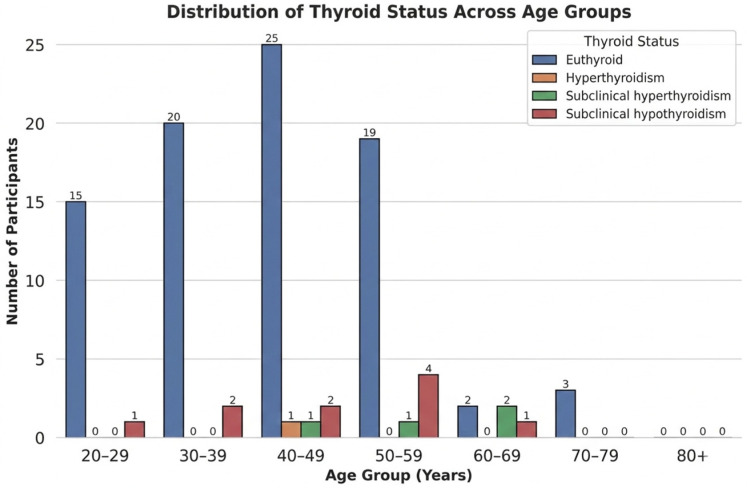
Distribution of thyroid function categories across different age groups. Euthyroid individuals were predominant in all age groups, particularly between 30 and 59 years. Subclinical hypothyroidism appeared more frequently in older participants, while subclinical and overt hyperthyroidism were relatively uncommon. This pattern reflects the age-related increase in mild thyroid dysfunction typically seen in clinical populations.

Age: The median age did not differ significantly among thyroid categories (*p* > 0.05), though participants with subclinical hypothyroidism tended to be slightly older on average. This aligns with epidemiologic reports indicating that mild thyroid failure increases with age (Figure 5).

Gender: Female patients predominated across all categories, in particular hypothyroid and subclinical groups, a pattern consistent with the known female predisposition to autoimmune thyroid dysfunction (Table 1).

Body Mass Index: BMI showed some variation across different thyroid function categories, but these differences were not statistically significant (*p* > 0.05). Still, the descriptive data revealed a trend toward higher BMI among participants with elevated TSH levels—mainly those in the euthyroid or subclinical hypothyroid range—compared to those with hyperthyroidism. This pattern is consistent with previous studies suggesting that higher body mass is often associated with mildly increased TSH levels.

#### 3.5.3. Association with Ultrasound Findings

The number of nodules did not differ significantly between thyroid function groups (*p* > 0.05). Nodules appeared in patients of almost all categories, suggesting that they are not only related to hormonal changes, but may also reflect structural variations in the thyroid gland itself. Similarly, TI-RADS scores showed no clear differences between the thyroid functional states (*p* > 0.05). However, patients with hyperthyroid or subclinical hyperthyroid status showed trends for slightly higher TI-RADS scores. This may indicate the presence of hyperfunctioning nodules; however, this pattern was not statistically significant. Overall, no statistically significant associations were identified between TI-RADS classification and thyroid hormone levels in the present sample. However, given the sample size, the presence of undetected small effects cannot be excluded.

### 3.6. Multivariable Regression

In adjusted regression models (*n* = 90), TI-RADS category was not independently associated with log-transformed TSH (overall TI-RADS *p* = 0.60) or FT4 (overall TI-RADS *p* = 0.105) after controlling for age, sex, and BMI. BMI remained positively associated with log-transformed TSH (β = 0.0277 per kg/m^2^, *p* = 0.018), whereas age and sex were not consistently associated with FT4 (Table 5).

Overall, BMI demonstrated the strongest physiological relationship with thyroid hormone markers, while US-based structural measures (TI-RADS and nodule count) were not significantly correlated with TSH or FT4 levels.

## 4. Discussion

Thyroid disorders are a major global health issue with a steadily rising prevalence over the past two decades. Recent reports suggest that nearly one in five adults may have some type of thyroid dysfunction, ranging from mild to more serious forms of hypo- or hyperthyroidism [16]. The increased incidence has been attributed to improved diagnostic sensitivity, environmental exposure to endocrine disruptors, and aging populations [2,3,4]. Epidemiological patterns in Saudi Arabia mirror global trends, but show higher female predominance and increased rates of subclinical hypothyroidism [14]. Increasing rates and subclinical manifestations underscore the importance of integrating biochemical and imaging tools for early detection and management.

US is well established as the primary clinical method for assessing thyroid morphology. Its sensitivity and non-invasive application make it an essential complement to clinical and laboratory evaluations [17]. High-resolution sonography allows accurate measurement of thyroid size, delineation of parenchymal texture, and identification of small nodules undetectable by palpation. Systems such as TI-RADS enable standardized malignancy-risk stratification, guiding biopsy decisions and follow-up strategies [10]. Normal thyroid tissue typically appears homogeneous and moderately echogenic relative to adjacent neck muscle; abnormal findings such as hypoechogenicity, microcalcifications, or irregular margins suggest inflammatory or neoplastic changes. Nodule size remains clinically relevant, as lesions exceeding 10 mm with suspicious sonographic features often warrant cytological evaluation [7].

Most participants in our study had euthyroid or subclinical hypothyroid function, with predominantly benign-appearing nodules categorized as TI-RADS 2–3. This distribution aligns with prior population-based research reporting that 60–70% of thyroid nodules detected by ultrasound are benign [5,18]. Although we observed no significant correlation between TSH or FT4 levels and TI-RADS categories, there was a subtle trend toward higher TI-RADS grades in participants with mildly elevated TSH. This observation is consistent with earlier studies suggesting a link between elevated serum TSH and an increased risk of malignancy [9]. To reduce potential confounding, multivariable regression models were adjusted for age, sex, and BMI, factors known to influence thyroid hormone regulation and nodule prevalence. The inclusion of these covariates did not meaningfully affect the lack of association between TI-RADS classification and thyroid hormone levels; this indicates that the observed independence between structural imaging findings and biochemical parameters was not attributable to these demographic or metabolic variables.

In the multivariable regression analysis, male sex was independently associated with lower log-transformed TSH levels after adjustment for age and BMI. This finding is consistent with epidemiological data demonstrating a higher prevalence of elevated TSH and thyroid dysfunction among women, particularly in relation to autoimmune thyroid disorders [1,12,19]. The observed association likely reflects established sex-related biological and immunological differences rather than a direct causal influence of sex itself. However, given the predominance of female participants in this cohort, this result should be interpreted cautiously. In contrast, no significant adjusted predictors of FT4 were identified. This may indicate that FT4 levels remained relatively stable across demographic and structural variables in this sample, potentially reflecting tighter physiological regulation of circulating thyroid hormone compared to the more sensitive pituitary feedback response reflected by TSH [1,16].

Nevertheless, BMI demonstrated a statistically significant but modest association with thyroid hormone levels, showing a small positive correlation with TSH and a small negative correlation with FT4 (ρ = −0.20). However, the magnitude of these associations was small and accounted for only a minor proportion of the variability in hormone levels. Accordingly, while statistically significant, the clinical relevance of BMI in relation to thyroid function within this cohort appears limited. These findings align with previous reports indicating subtle alterations in the hypothalamic–pituitary–thyroid axis in individuals with higher levels of adiposity, possibly mediated by leptin signaling or chronic low-grade inflammation [19]. However, the small effect size we observed suggests that BMI is a contributing factor to, rather than a primary driver of, variability in thyroid hormone levels. The observed correlation (ρ = 0.20) corresponds to approximately 4% small effect, reinforcing that body mass accounts for only a small fraction of hormonal variability.

The lack of a statistically significant association between thyroid hormone levels and TI-RADS classification has important clinical implications. Specifically, it indicates that biochemical euthyroidism does not exclude the presence of structurally suspicious nodules; US-based risk assessment cannot be inferred from hormone levels alone. In clinical practice, a reliance on thyroid function tests alone may fail to identify patients with morphologically high-risk nodules. These findings support the view that laboratory evaluation and imaging assessment serve complementary, rather than interchangeable, roles in thyroid nodule evaluation.

The absence of a strong link between thyroid hormone levels and US findings suggests that hormonal and structural changes in the thyroid may progress independently, particularly during the early stages of disease. FNA results from a subset of patients showed an almost significant trend by which higher TI-RADS grades were associated with abnormal cytology findings. This pattern is consistent with earlier regional findings [11], suggesting that TI-RADS retains moderate predictive validity even in heterogeneous endocrine populations. However, the lack of statistical significance—likely due to the small number of patients who underwent FNA in this sample—emphasizes the need for larger studies to confirm these associations. The trend between higher TI-RADS categories and abnormal cytology warrants further investigation in larger samples. These findings highlight that thyroid nodule morphology may not directly mirror hormonal function, reinforcing the need for integrated assessment using both imaging and laboratory parameters.

Overall, our findings provide valuable insight from a Saudi population. They show an expected close link between BMI and thyroid hormone levels, while confirming that US findings—specifically TI-RADS classifications—do not always match hormonal status. Clinically, this means that normal hormone levels cannot rule out structural thyroid abnormalities, highlighting the need for both biochemical and imaging assessments. In addition, these results underline the importance of local data, since the pattern and frequency of thyroid disorders in Saudi Arabia are likely affected by regional iodine intake and demographic characteristics.

In summary, this study adds valuable insight to the current evidence on thyroid dysfunctions by combining US findings with hormone measurements in real clinical cases. While our results did not show a significant relationship between hormone levels and TI-RADS scores, the observed patterns highlight the complex nature of thyroid regulation. These findings indicate the value of integrating laboratory testing with standardized US assessment to provide a clearer and more complete understanding of thyroid function.

## 5. Limitations

This study has several limitations that should be acknowledged. First, its retrospective, cross-sectional design limits our ability to infer causality between thyroid hormone levels and US-based structural changes. We observed associations, but we cannot determine whether hormonal fluctuations precede or follow nodular development. A prospective follow-up would be more informative in clarifying this temporal relationship.

Second, although the sample size (*n* = 102) was sufficient for descriptive and exploratory analyses, it was relatively small, and FNA results were only available from a limited number of participants. This may have limited our ability to detect small-to-moderate associations between thyroid hormone levels, cytological findings, and TI-RADS categories. Based on an alpha level of 0.05 and a two-tailed test, a sample of this size provides approximately 80% statistical power to identify correlations of r = 0.27 or higher. Consequently, weaker associations may have gone undetected, raising the possibility of a type II error. Future studies involving larger, multicenter cohorts are required to determine whether subtle relationships exist between biochemical thyroid parameters and US-based risk stratification.

Although TI-RADS classification was performed by experienced radiologists using standardized ACR criteria, interobserver agreement was not formally assessed. Therefore, some degree of variability in US interpretation cannot be excluded. The data were also collected from a single tertiary hospital in Tabuk, which may not fully reflect the broader Saudi population. Regional factors such as iodine intake, genetic background, and environmental exposures could all influence thyroid function and structure.

Third, some potentially important confounding factors—including iodine status and medication history—were not consistently recorded in the hospital database. In addition, data on thyroid autoimmunity markers, including anti-TPO and anti-Tg antibodies, were not consistently available. This limitation restricts our ability to determine whether the relatively high prevalence of subclinical hypothyroidism in this cohort is attributable to underlying autoimmune thyroiditis, iodine-related factors, or other metabolic influences. Future studies that incorporate comprehensive autoimmune profiling would help clarify the underlying causes of mild thyroid dysfunction in this population.

Despite these limitations, this study provides a useful reflection of real clinical practice and adds valuable local data that complement larger international research. The close agreement between our findings and those from other studies strengthens the credibility of our results. Together, these data underscore the importance of combining laboratory tests with imaging evaluation in routine thyroid assessment.

## 6. Future Work

Future studies should continue this work in several directions. A larger, prospective study that includes several hospitals across Saudi Arabia would help represent the wider population and confirm whether similar trends appear in areas with different iodine levels and demographics. Including tests for thyroid autoimmune markers alongside imaging and hormonal parameters would allow a more comprehensive evaluation of the interplay between thyroid autoimmunity, structural risk stratification, and functional status.New imaging tools may also improve thyroid disorder diagnosis. Techniques like elastography, quantitative US, or even AI-based image analysis could help identify nodules that are more likely to be malignant beyond current TI-RADS capabilities. Using machine learning with combined data—such as hormones, imaging, and patient characteristics—could reveal subtle patterns that are difficult for clinicians to identify.

It would also be valuable to follow euthyroid patients who exhibit structural thyroid changes. Longitudinal observation of hormonal and structural progression over time could clarify whether early biochemical changes predict later structural problems. Linking imaging results with patient outcomes—such as symptom changes, surgery, or biopsy results—would provide clinically meaningful information.

By exploring these directions, future research can move closer to a clearer and more practical model of thyroid health, connecting functional and structural aspects in a way that helps clinicians make better decisions for their patients.

## 7. Conclusions

In conclusion, this study shows the complex relationship between thyroid function and structural changes observed on US. Although we did not find a clear correlation between TSH, FT4, and TI-RADS categories, some patterns suggest that hormonal and structural changes may develop separately. BMI showed a mild association with thyroid hormone levels, indicating metabolic influence on thyroid regulation. These findings highlight the importance of evaluating both laboratory and imaging results together to provide a more complete picture of thyroid health. By presenting data from a Saudi population, this study adds valuable local insight to global research and emphasizes the need for a multidisciplinary approach in diagnosing and managing thyroid disorders.

## Figures and Tables

**Table 1:** Mean body mass index (BMI) of participants by age group and gender (*n* = 102). Across all age groups, females generally had higher mean BMI than males, with middle-aged (50–59 years) participants of both sexes showing increased BMI, with a gradual decline in older groups. This trend reflects the typical midlife weight gain pattern observed in thyroid clinic populations.

Age Group (*n* = 102)	Female Mean BMI ± SD	Male Mean BMI ± SD
20–29	24.00 ± 4.19	18.25 ± — *
30–39	28.60 ± 5.59	25.48 ± 4.11
40–49	27.16 ± 5.99	27.80 ± 4.06
50–59	33.62 ± 8.90	36.46 ± 7.42
60–69	29.76 ± 5.31	27.94 ± 0.51
70–79	26.31 ± — *	26.03 ± 0.53

SD not calculated (*) where *n* = 1 (insufficient degrees of freedom).

**Table 2:** Distribution of fine-needle aspiration (FNA) cytology results across Thyroid Imaging Reporting and Data System (TI-RADS) categories. Most nodules classified as TI-RADS 2 or 3 were cytologically benign; malignant or suspicious lesions appeared only in TI-RADS 4. A small number of cases fell into the “other” category, indicating indeterminate or non-diagnostic samples. This pattern supports the general reliability of TI-RADS in reflecting cytological risk.

FNA Category	2	3	4
Benign	3	3	0
Malignant/Suspicious	0	0	2
Other	2	7	5

**Table 3:** Thyroid function classification criteria based on international reference ranges for thyroid-stimulating hormone (TSH) and free thyroxine (FT4). Participants were categorized into five groups representing normal, subclinical, and overt thyroid function. The classification follows global endocrine guidelines, where deviations in TSH and FT4 levels help differentiate between biochemical and clinically evident thyroid dysfunction.

Category	TSH (mIU/L)	FT4 (pmol/L)	Description
Euthyroid	0.4–4.0	10.3–23.2	Normal thyroid function
Subclinical Hypothyroidism	>4.0	10.3–23.2	Elevated TSH with normal FT4
Subclinical Hyperthyroidism	<0.4	10.3–23.2	Suppressed TSH with normal FT4
Overt Hypothyroidism	>4.0	<10.3	Elevated TSH with low FT4
Overt Hyperthyroidism	<0.4	>23.2	Suppressed TSH with high FT4

**Table 4:** Distribution of participants across thyroid function categories according to standard thyroid-stimulating hormone (TSH) and free thyroxine (FT4) ranges. Most individuals were euthyroid or had subclinical hypothyroidism, while overt thyroid dysfunctions were relatively uncommon.

Thyroid Function Category	Frequency (*n*)	Percentage (%)	Interpretation
Euthyroid	59	58.3%	Most participants had normal thyroid function.
Subclinical Hypothyroidism	28	27.8%	Mild thyroid failure was common, particularly in older females.
Subclinical Hyperthyroidism	8	7.4%	Relatively uncommon; often asymptomatic.
Overt Hypothyroidism	5	4.6%	Low FT4 with elevated TSH; few cases detected.
Overt Hyperthyroidism	2	1.9%	Rare in this cohort; characterized by suppressed TSH and elevated FT4.
Total	102	100%	

**Table 5:** In multivariable analyses adjusted for age, sex, and body mass index (BMI), Thyroid Imaging Reporting and Data System (TI-RADS) category was not independently associated with log-transformed TSH or FT4 levels. BMI remained positively associated with log-transformed TSH (β = 0.0277 per kg/m^2^, *p* = 0.018). Male sex was independently associated with lower log-transformed TSH values (β = −0.819, *p* = 0.024). No significant adjusted predictors of FT4 were identified.

Predictor	Log-Transformed (TSH) β (95% CI)	*p*-Value	FT4 β (95% CI)	*p*-Value
TI-RADS Category				
TI-RADS 3 vs. 2	0.296 (−0.335 to 0.926)	0.358	0.016 (−1.340 to 1.371)	0.982
TI-RADS 4 vs. 2	0.274 (−0.276 to 0.824)	0.329	−0.951 (−2.055 to 0.153)	0.091
Age (years)	0.002 (−0.007 to 0.011)	0.677	−0.010 (−0.039 to 0.019)	0.505
Male sex (vs female)	−0.819 (−1.530 to −0.109)	0.024 *	−0.342 (−1.507 to 0.822)	0.563
BMI (kg/m^2^)	0.0277 (0.0048 to 0.0506)	0.018 *	−0.039 (−0.098 to 0.020)	0.191

* Statistically significant at *p* < 0.05 (two-tailed).

## Data Availability

The dataset used in this study is derived from hospital electronic medical records and contains sensitive clinical information. Therefore, it is not publicly available. De-identified data may be made available from the corresponding author upon reasonable request, subject to institutional approvals and data-sharing regulations.
